# Supplementary material for: Angicin, a novel bacteriocin of Streptococcus anginosus
Source: Sci Rep. 2021 Dec 21;11:24377. doi: 10.1038/s41598-021-03797-5 (PMC8692603; doi:10.1038/s41598-021-03797-5)
Supplement: Supplementary file 1 — Supplementary Information. [file 41598_2021_3797_MOESM1_ESM.docx]

Supplementary Information

Verena Vogel^1^, Richard Bauer^1^, Stefanie Mauerer^1^, Nicole Schiffelholz^2^, Christian Haupt^2^, Gerd M. Seibold^3^, Marcus Fändrich^2^, Paul Walther^4^ and Barbara Spellerberg^1^

^1^ Institute of Medical Microbiology and Hygiene, Ulm University Medical Center, Ulm, Germany

^2^  Institute of Protein Biochemistry, Ulm University, 89081 Ulm, Germany

^3^ Department of Biotechnology and Biomedicine, Technical University of Denmark, Kongens Lyngby, Denmark

^4^ Central Facility for Electron Microscopy, Ulm University, Ulm, Germany

* Correspondence: barbara.spellerberg@uniklinik-ulm.de; Tel.: +49 731 500 65333

Content

[Figure S1: Genetic alignment of amino acid sequences of Angicin prepetide and homologs. 3](#_Toc88058751)

[Figure S2: Activity of cell free supernatant. 4](#_Toc88058752)

[Figure S3: Growth inhibition of *Listeria monocytogenes* by CFS treatment. 5](#_Toc88058753)

[Figure S4: Effect of recombinantly expressed Blp3.3 on antimicrobial activity. 6](#_Toc88058754)

[Table S1: Screen of *Streptococcus anginosus* isolates for bacteriocin production. 7](#_Toc88058755)

[Table S2: Minimal inhibition concentration (MIC) of Angicin. 10](#_Toc88058756)

[Table S3: Bacterial strains and plasmids used in this study 11](#_Toc88058757)

[Table S4: Primers used in this study 14](#_Toc88058758)

[References 16](#_Toc88058759)


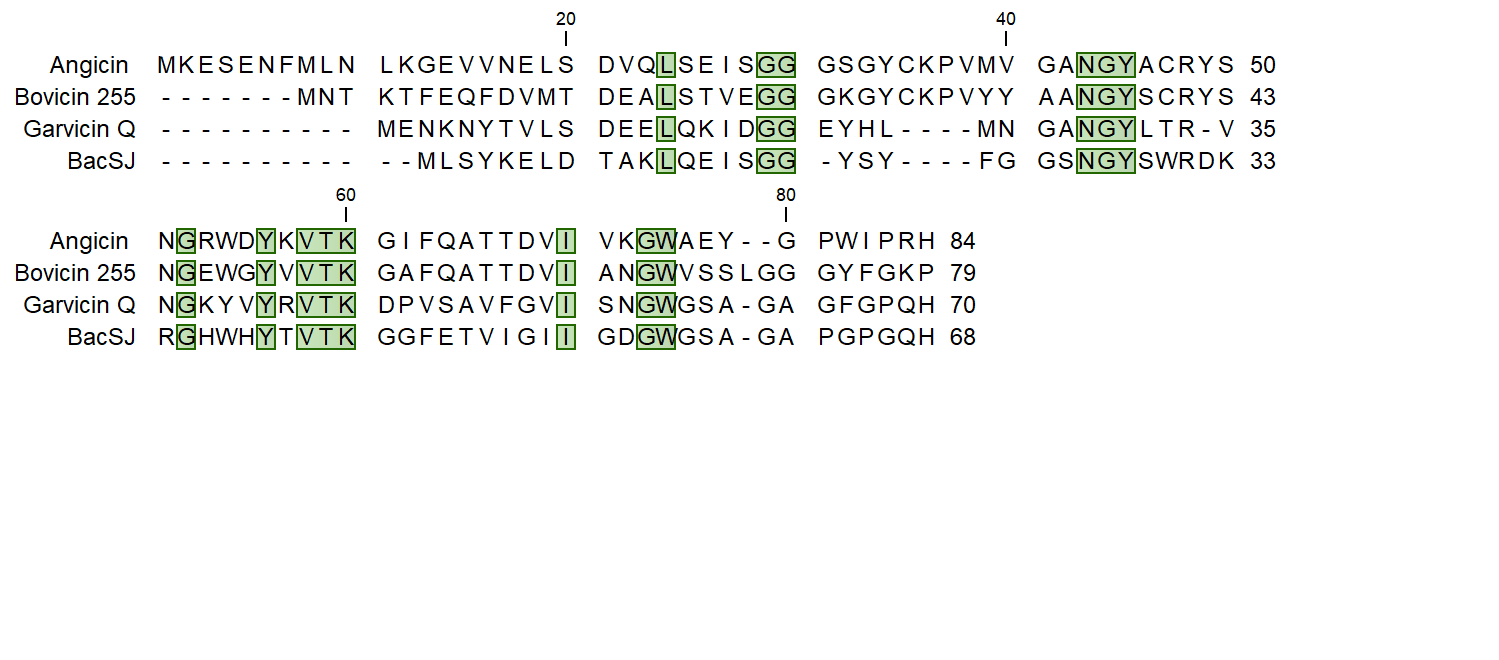


# **Figure S1: Genetic alignment of amino acid sequences of Angicin prepetide and homologs**.

Angicin prepetide was compared to Bovicin variant 255 (GenBank: AAG29818.1), Garvicin Q (GenBank: AEN79392.1) and BacSJ (GenBank: CAR92206.2) using CLC main workbench V7. Conserved residues are marked in green.





**Figure S2: Activity of cell free supernatant.**

Activity of cell free supernatant (CFS) was assessed in a two-layer RDA against *Listeria monocytogenes* and compared to the activity of *Streptococcus anginosus* BSU 1211 in a one-layer radial diffusion assay.

**

**

**Figure S3: Growth inhibition of *Listeria monocytogenes* by CFS treatment.**

*L. monocytogenes* was incubated with either cell free supernatant (CFS) of BSU 1211, CFS of BSU 1211∆*blp3* or no CFS over a time course of 9 h. Each hour growth was measured via absorbance at 600 nm in a Tecan plate reader. Data was collected in at least five independent experiments. A significant difference between cells treated with CFS of BSU 1211 or cells treated with CFS of BSU 1211∆*blp3* was tested with a Mann-Whitney-U-test (* indicates p<0.05, ** indicates p<0.01 and *** indicates p<0.001).


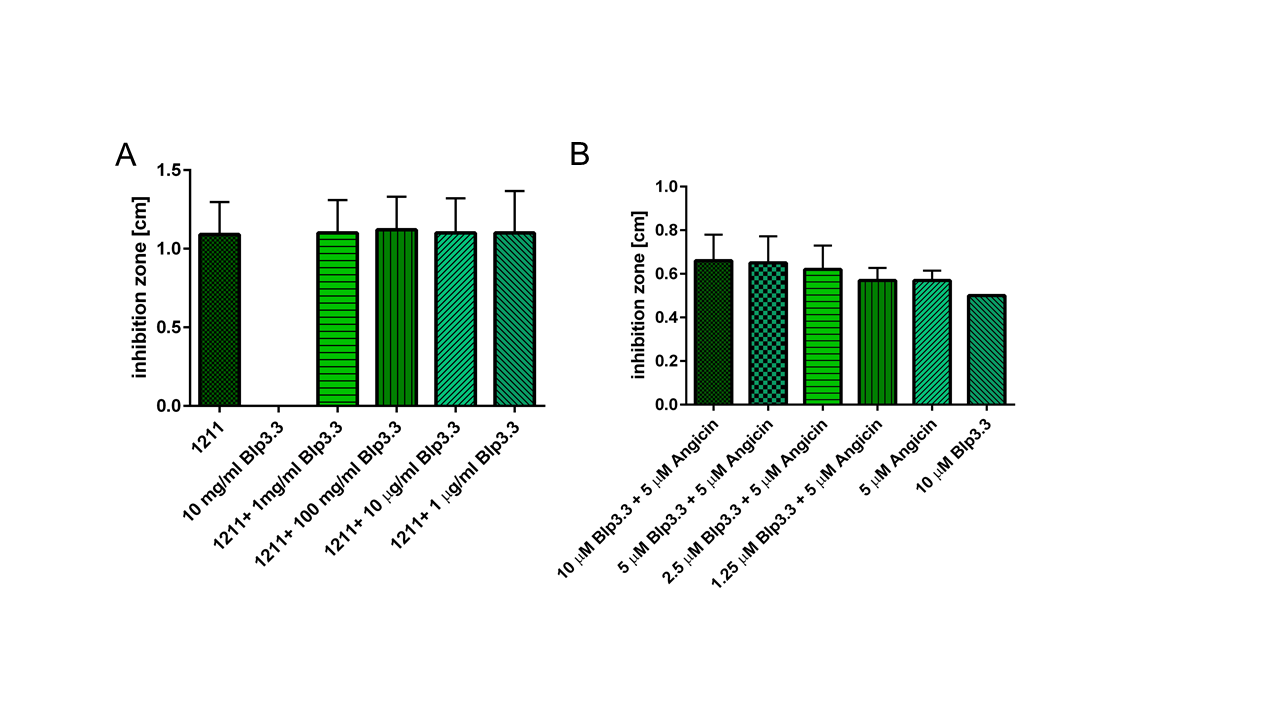


**Figure S4: Effect of recombinantly expressed Blp3.3 on antimicrobial activity.**

(A) Different Blp3.3 concentrations were simultaneously added with BSU 1211 in a one-layer RDA against *L. monocytogenes.* After overnight incubation inhibition zone size was analyzed. Blp3.3 alone was used as a control. (B) Angicin was preincubated with indicated Blp3.3 concentrations for 1 h at 37 °C. Afterwards the antimicrobial activity against *L. monocytogenes* was surveyed in a two-layer RDA. Depicted are five independent experiments conducted with three technical replicates. With a Mann-Whitney-U-test it was controlled for significant differences to either the wildtype or to Angicin alone.

# Table S1: Screen of *Streptococcus anginosus* isolates for bacteriocin production.

*S. anginosus* SK52, *Streptococcus intermedius, Streptococcus constellatus, Streptococcus pyogenes* were used as target strains in a one-layer radial diffusion assay and tested for susceptibility towards clinical *S. anginosus* isolates. Inhibition zone size measured in one experiment is depicted in cm.

| *Streptococcus anginosus* clinical isolate (BSU number) | *Streptococcus anginosus* SK52 | *Streptococcus intermedius* | *Streptococcus constellatus* | *Streptococcus pyogenes* |
| --- | --- | --- | --- | --- |
| 1210 | 0 | 0 | 0 | 0.5 |
| 1211 | 0.55 | 0.2 | 1.3 | 0.1 |
| 1212 | 0 | 0 | 0 | 0.2 |
| 1214 | 0 | 0.6 | 0 | 0.5 |
| 1215 | 0 | 0 | 0.2 | 0 |
| 1216 | 0 | 0 | 0 | 0.2 |
| 1217 | 0 | 1 | 0 | 0.2 |
| 1222 | 0 | 0 | 0 | 0 |
| 1227 | 0 | 0 | 0 | 0 |
| 1289 | 0 | 0 | 0 | 0 |
| 1292 | 0 | 0 | 0 | 0 |
| 1303 | 0 | 0 | 0 | 0.5 |
| 1304 | 0.2 | 0.5 | 0.4 | 0.7 |
| 1306 | 0 | 0 | 0 | 0.2 |
| 1307 | 0 | 0.4 | 0.2 | 0.5 |
| 1308 | 0 | 0 | 0 | 0 |
| 1310 | 0 | 0.5 | 0 | 0.5 |
| 1312 | 0 | 0 | 0 | 0 |
| 1313 | 0.2 | 0.4 | 0.2 | 0 |
| 1317 | 0 | 0.4 | 0 | 0 |
| 1318 | 0 | 0.2 | 0 | 0 |
| 1319 | 0 | 0.4 | 0.3 | 0.2 |
| 1323 | 0 | 0 | 0 | 0 |
| 1324 | 0 | 0.2 | 0 | 0 |
| 1326 | 0 | 0 | 0 | 0 |
| 1327 | 0 | 0 | 0 | 0 |
| 1328 | 0 | 0 | 0 | 0 |
| 1329 | 0 | 0 | 0 | 0 |
| 1330 | 0.2 | 0.4 | 0.3 | 0.5 |
| 1331 | 0 | 0.2 | 0.2 | 0.4 |
| 1332 | 0 | 0.2 | 0 | 0 |
| 1334 | 0 | 0.3 | 0 | 0.4 |
| 1336 | 0 | 0.6 | 0 | 0 |
| 1338 | 0 | 0.4 | 0 | 0 |
| 1339 | 0 | 0.6 | 0 | 0 |
| 1344 | 0 | 0.5 | 0 | 0 |
| 1345 | 0 | 0.4 | 0 | 0 |
| 1346 | 0 | 0.3 | 0 | 0 |
| 1351 | 0 | 0 | 0 | 0.2 |
| 1354 | 0.2 | 0.5 | 0 | 0.2 |
| 1355 | 0 | 0 | 0 | 0 |
| 1356 | 0 | 0 | 0 | 0 |
| 1358 | 0 | 0 | 0 | 0.2 |
| 1360 | 0 | 0.3 | 0 | 0.2 |
| 1361 | 0 | 0 | 0 | 0.5 |
| 1362 | 0 | 0 | 0 | 0 |
| 1363 | 0 | 0 | 0 | 0 |
| 1364 | 0 | 0 | 0 | 0 |
| 1365 | 0 | 0 | 0 | 0 |
| 1366 | 0.5 | 0 | 0 | 0 |
| 1367 | 0 | 0 | 0 | 0 |
| 1369 | 0 | 0 | 0 | 0.2 |
| 1370 | 0.2 | 0 | 1 | 0 |
| 1372 | 0 | 0 | 0 | 0.2 |
| 1373 | 0 | 0 | 0 | 0.5 |
| 1376 | 0 | 0 | 0 | 0 |
| 1379 | 0 | 0 | 0 | 0 |
| 1381 | 0 | 0 | 0 | 0 |
| 1382 | 0 | 0 | 0 | 0 |
| 1384 | 0 |  |  | 0.4 |
| 1386 | 0 | 0.2 | 0.5 | 0 |
| 1387 | 0.6 | 0.9 | 0.7 | 0.5 |
| 1388 | 0 | 0.2 | 0 | 0.2 |
| 1389 | 0.2 | 0.9 | 0 | 0 |
| 1390 | 0 | 0.7 | 0 | 0.2 |
| 1391 | 0 | 0.2 | 0 | 0 |
| 1392 | 0 | 0 | 0 | 0 |
| 1395 | 0 | 0 | 0.2 | 0 |
| 1396 | 0 | 0 | 0 | 0 |
| 1397 | 0 | 0 | 0 | 0 |
| 1398 | 0 | 0 | 0 | 0 |
| 1399 | 0 | 0 | 0 | 0.2 |
| 1400 | 0 | 0 | 0 | 0 |
| 1401 | 0 | 0 | 0 | 0 |
| 1402 | 0 | 0.5 | 0 | 0 |
| 1403 | 0 | 0.2 | 0 | 0.2 |
| 1404 | 0 | 0 | 0 | 0 |
| 1405 | 0 | 0.4 | 0 | 0 |
| 1406 | 0 | 0 | 0 | 0 |
| 1407 | 0 | 0 | 0 | 0 |
| 1408 | 0 | 0 | 0 | 0 |
| 1409 | 0.2 | 0 | 0.5 | 0.4 |
| 1410 | 0 | 0 | 0 | 0 |
| 1411 | 0 | 0 | 0 | 0 |
| 1412 | 0 | 0 | 0 | 0 |
| 1413 | 0 | 0 | 0 | 0 |
| 1414 | 0 | 0 | 0 | 0 |
| 1415 | 0 | 0 | 0 | 0 |
| 1416 | 0 | 0 | 0 | 0 |
| 1417 | 0 | 0 | 0 | 0 |
| 1418 | 0 | 0.2 | 0 | 0.4 |
| 1419 | 0 | 0.6 | 0 | 0.5 |
| 1420 | 0 | 0 | 0 | 0 |
| 1421 | 0 | 0 | 0 | 0 |
| 1422 | 0 | 0 | 0 | 0 |

Table S2: Minimal inhibition concentration (MIC) of Angicin.

Growth of sensitive species after 24 h incubation with varying Angicin concentrations was determined by absorbance measurement.

| Species | MIC |
| --- | --- |
| *L. monocytogenes* | 3.125 µg/ml |
| *L. grayi* | 3.125 µg/ml |
| *L. ivanovii* | > 100 µg/ml |
| VRE | > 100 µg/ml |

Table S3: Bacterial strains and plasmids used in this study

| **Strain or plasmid** | **Definition** | **Source** |
| --- | --- | --- |
| *Escherichia coli* DH5α | *endA1 hsdR17 supE44* DlacU169(f80lacZDM15) *recA1 gyrA96 thi-1 relA1* | Boehringer |
| *E. coli* EC101 | E. coli JM101 derivative with *repA* from pWV01 integrated into the chromosome | ^1^ |
| *Streptococcus anginosus* BSU 1211^a^ | *S. anginosus*, clinical isolate | ^2^ |
| *S. anginosus* BSU 1324^a^ | *S. anginosus*, clinical isolate | ^2^ |
| *S. anginosus* BSU 1370^a^ | *S. anginosus*, clinical isolate | This study |
| *S. anginosus* BSU 1401^a^ | *S. anginosus*, clinical isolate | ^2^ |
| *S. anginosus* SK 52 | *S. anginosus* type strain, ATCC 33397, Hly+ | ATCC |
| *Streptococcus constellatus* BSU 1213^a^ | *S. constellatus*, clinical isolate | This study |
| *Streptococcus intermedius* BSU 1340^a^ | *S. intermedius*, clinical isolate | This study |
| *Streptococcus pyogenes* BSU 998^a^ | *S. pyogenes* type strain, ATCC 12344 | ATCC |
| *Listeria monocytogenes* EGDe^b^ (BSU 1423) | Ln II Serotype I/2a | ^3^ |
| *Listeria ivanovii* CIP 78.42T^b^ (BSU 1430) | - | ^4^ |
| *Listeria grayi* CIP 68.18T^b^ (BSU 1431) | - | ^4^ |
| *Streptococcus dysagalactiae subsp. equisimilis* BSU 226^c^ | Serotype C | This study |
| *Streptococcus dysagalactiae subsp. equisimilis* BSU 267^c^ | Serotype G | This study |
| *Streptococcus mutans* BSU 269 | DSM 20523 | DSM |
| *Streptococcus agalactiae* BSU 308 | ATCC 12403= NEM 316 | ATCC |
| *Streptococcus suis* BSU 320^d^ | Serotype 2 | This study |
| *Streptococcus porcinus* BSU 852^e^ | - | This study |
| *Streptococcus pneumoniae* BSU 994 | ATCC 49619 | ATCC |
| *Streptococcus mitis* BSU 999 | ATCC 49456 | ATCC |
| *Streptococcus oralis* BSU 1342^a^ | *S. oralis* clinical isolate | This study |
| *Bacillus subtilis* BSU 851 | ATCC 6633 | ATCC |
| *Pseudomonas aeroginosa* BSU 856 | ATCC 27853 | ATCC |
| *Staphylococcus aures* BSU 1348 | MRSA, ATCC 43300 | ATCC |
| *Klebsiella pneumoniae* BSU 1353 | ESBL, ATCC 7000603 | ATCC |
| *Enterococcus faecium* BSU 1516 | VRE, DSM 17050 | DSM |
| *Acinetobacter baumanni* BSU 1514 | *A. baumannii* type strain, ATCC 19606 | ATCC |
| *S. aureus* BSU 995 | ATCC 25923 | ATCC |
| *Staphylococcus epidermidis* BSU 993 | ATCC 12228 | ATCC |
| *S. aureus* BSU 857 | ATCC 29213 | ATCC |
| *S. aureus* BSU 878 | ATCC 13565 | ATCC |
| *Lactobacillus acidophilus* BSU 1314 | *L. acidophilus* | food isolate |
| *Lactobacillus gasseri* BSU 1315 | *L. gasseri* | food isolate |
| *Lactobacillus casei* BSU 1316 | *L. casei* | food isolate |
| *Lactobacillus rhamnosus* BSU 853^e^ | - | This study |
| *Lactobacillus paracasei* BSU 854^e^ | - | This study |
| **Mutants** |  |  |
| *S. anginosus* BSU 1211∆*blp3* |  | This study |
| *S. anginosus* BSU 1211∆*blp3* + pAT18_*blp3* |  | This study |
| *S. anginosus* BSU 1211∆*blp3* + pAT18 |  | This study |
| *S. anginosus* SK52 + pAT18_*blp3* |  | This study |
| *S. anginosus* BSU 1211∆*blp3.1* |  | This study |
| *S. anginosus* BSU 1211∆*blp3.4* |  | This study |
| *S. anginosus* BSU 1211∆*blp3.6* |  | This study |
| *S. anginosus* SK52 + pAT28_*blp3.3* |  | This study |
| *S. anginosus* SK52 + pAT28_*promblp3.3* |  | This study |
| *S. constellatus* BSU 1213 + pAT28_*blp3.3* |  | This study |
| *S. constellatus* BSU 1213 + pAT28_*promblp3.3* |  | This study |
| **Plasmids** |  |  |
| pAT18 | pAT18-lacZα, ori pUC, ori pAmβ1, Em^R^ | ^5^ |
| pAT18-*blp3* | pAT18 derivate carrying the complete *blp3* region, Em^R^ | This study |
| pAT18-cre-rec_tufA_ | pAT18 derivative carrying Cre-recombinase gene under the control of *tufA* promoter, Em^R^ | ^6^ |
| pAT28 | lacZα, ori pUC, ori pAmβ1, Spc^R^ | ^7^ |
| pAT28-EGFP_cfb_ | pAT28 derivate carrying EGFP gene under the control of *cfb* promotor, Spc^R^ | ^8^ |
| pAT28- *blp3.3* | pAT28 derivate carrying *blp3.3* gene, Spc^R^ | This study |
| pAT28- *blp3.3*_prom_ | pAT28 derivate carrying *blp3.3* gene under the control of an endogenous promotor, Spc^R^ | This study |
| pAT28- *blp3.3*_cfb_ | pAT28 derivate carrying *blp3.3* gene under the control of *cfb* promotor, Spc^R^ | This study |
| pGA14-Spc | Replication functions of pWVO1, Em^R^, Spc^R^ | ^9^ |

^a^ isolated at university hospital Ulm, Ulm, Germany

^b^ kindly provided by Prof. Dr. C. Riedel, Ulm University, Ulm, Germany

^c^ kindly provided by RWTH Aachen University, Aachen, Germany

^d^ kindly provided by University Hospital Düsseldorf, Düsseldorf, Germany

^e^ obtained from INSTAND e.V., Düsseldorf, Germany

Table S4: Primers used in this study

| Primer name | Sequence | Nr. |
| --- | --- | --- |
| Blp3_screen1_fwd | tttcataatgttcccgattg | 1 |
| Blp3_screen1_rev | agtatgttaatcgctctaatc | 2 |
| Blp3_screen2_fwd | gggcacagtattgtacagg | 3 |
| Blp3_screen2_rev | tccctaataactagctctac | 4 |
| Blp3_screen3_rev | gtagctctgatagcctgaatg | 5 |
| Blp3.3_fwd | tggtgggcaggaaagaag | 6 |
| Blp3.3_rev | caccctcccagatgttatcg | 7 |
| Sil_screen1_fwd | catagcctctatctggtatatc | 8 |
| Sil_screen1_rev | cgtaaatgacggtctaaataattgg | 9 |
| Sil_screen2_fwd | caattatggcgacgctgatag | 10 |
| Sil_screen2_rev | gcggcgacttatgacaatag | 11 |
| Sil_screen3_fwd | ctgtttcgggagcgactaatc | 12 |
| Sil_screen3_rev | caaatccgtcttaatggaaatg | 13 |
| Sil_screen4_fwd | caatttcacaagcgcgaataatc | 14 |
| Sil_screen4_rev | gtattctactaaccggctttgc | 15 |
| SilCR_1211_fwd | caactatgacgattgcttatg | 16 |
| SilCR_1211_rev | gcagtccatcaccattatc | 17 |
| Blp3.4_screen_fwd | ctttcctgcccaccagcaaac | 18 |
| Blp3.4_screen_rev | gattagagcgattaacatactaaag | 19 |
| Blp3.3_1211_EcoRI_fwd | gggcccgaattcatgggaactttaagatgg | 20 |
| Blp3.3_1211_prom_EcoRI_fwd | gggcgcgaattctgtcgctatagtaatggc | 21 |
| Blp3.3_1211_BamHI_rev | ccgggcggatccctagattattctaatatgag | 22 |
| Blp3_1211_F1_fwd | aacgagctgtcttcctgtc | 23 |
| Blp3_1211_F1_rev | gcatacattatacgaacggtacgggtattgctggtggtg | 24 |
| Blp3_1211_F2_fwd | tataatgtatgctatacgaacggtacgactatgaataccaaac | 25 |
| Blp3_1211_F2_rev | aggattggaagttcacaag | 26 |
| lox71_spec_fwd | taccgttcgtatagcatacattatacgaagttatttaaatggcattggtaccc | 27 |
| lox66_spec_rev | taccgttcgtataatgtatgctatacgaagttatatgcctgcaggtcgattttcg | 28 |
| Blp3.1_del_F1_rev | tataatgtatgctatacgaacggtagagtagttgcaggagctgttg | 29 |
| Blp3.1_del_F2_fwd | gcatacattatacgaacggtacacttgccagcatttcagag | 30 |
| Blp3.1_del_F2_rev | ggctggtctgaatccttctac | 31 |
| Blp3.4_del_F1_rev | tataatgtatgctatacgaacggtagaatatggtccgtggattccaag | 32 |
| Blp3.4_del_F2_fwd | gcatacattatacgaacggtagaacatctgatagctcatttacaac | 33 |
| Blp3.6_del_F1_fwd | ctgatagctcatttacaacttc | 34 |
| Blp3.6_del_F1_rev | tataatgtatgctatacgaacggtagggcacagtattgtacagg | 35 |
| Blp3.6_del_F2_fwd | gcatacattatacgaacggtacacttgccagcatttcagag | 36 |
| Blp3.6_del_F2_rev | gaagaacagcatcaatatctc | 37 |
| Blp3.1_del_screen_fwd | cagttatagctgtgcgttgatg | 38 |
| Blp3.1_del_screen_rev | gaagctgaacaacctgattgg | 39 |
| Blp3.4_del_screen_fwd | ccaatcaggttgttcagcttctc | 40 |
| Blp3.4_del_screen_rev | cagtagcaggtggaacgatag | 41 |
| Blp3.6_del_screen_fwd | ctttcctgcccaccagcaaac | 42 |
| Blp3.6_del_screen_rev | gctgcgatatcacgtttatgg | 43 |
| Blp3_1211_EcoRI_fwd | gggcccgaattccaaacgagctgtcttcctg | 44 |
| Blp3_1211_BamHI_rev | ggccgcggatccctgataacactttatagc | 45 |

References

(1) Law, J.; Buist, G.; Haandrikman, A.; Kok, J.; Venema, G.; Leenhouts, K. A System to Generate Chromosomal Mutations in Lactococcus Lactis Which Allows Fast Analysis of Targeted Genes. *J. Bacteriol.* **1995**, *177* (24), 7011–7018.

(2) Bauer, R.; Neffgen, N.; Grempels, A.; Furitsch, M.; Mauerer, S.; Barbaqadze, S.; Haase, G.; Kestler, H.; Spellerberg, B. Heterogeneity of Streptococcus Anginosus SS-Hemolysis in Relation to CRISPR/Cas. *Mol. Oral Microbiol.* **2020**, *35* (2), 56–65. https://doi.org/10.1111/omi.12278.

(3) Bécavin, C.; Bouchier, C.; Lechat, P.; Archambaud, C.; Creno, S.; Gouin, E.; Wu, Z.; Kühbacher, A.; Brisse, S.; Pucciarelli, M. G.; García-del Portillo, F.; Hain, T.; Portnoy, D. A.; Chakraborty, T.; Lecuit, M.; Pizarro-Cerdá, J.; Moszer, I.; Bierne, H.; Cossart, P. Comparison of Widely Used Listeria Monocytogenes Strains EGD, 10403S, and EGD-e Highlights Genomic Variations Underlying Differences in Pathogenicity. *mBio* **2014**, *5* (2), e00969-00914. https://doi.org/10.1128/mBio.00969-14.

(4) Zetzmann, M.; Okshevsky, M.; Endres, J.; Sedlag, A.; Caccia, N.; Auchter, M.; Waidmann, M. S.; Desvaux, M.; Meyer, R. L.; Riedel, C. U. DNase-Sensitive and -Resistant Modes of Biofilm Formation by Listeria Monocytogenes. *Front. Microbiol.* **2015**, *6*, 1428. https://doi.org/10.3389/fmicb.2015.01428.

(5) Trieu-Cuot, P.; Carlier, C.; Poyart-Salmeron, C.; Courvalin, P. Shuttle Vectors Containing a Multiple Cloning Site and a LacZ Alpha Gene for Conjugal Transfer of DNA from Escherichia Coli to Gram-Positive Bacteria. *Gene* **1991**, *102* (1), 99–104. https://doi.org/10.1016/0378-1119(91)90546-n.

(6) Bauer, R.; Mauerer, S.; Grempels, A.; Spellerberg, B. The Competence System of Streptococcus Anginosus and Its Use for Genetic Engineering. *Mol. Oral Microbiol.* **2017**. https://doi.org/10.1111/omi.12213.

(7) Trieu-Cuot, P.; Carlier, C.; Poyart-Salmeron, C.; Courvalin, P. A Pair of Mobilizable Shuttle Vectors Conferring Resistance to Spectinomycin for Molecular Cloning in Escherichia Coli and in Gram-Positive Bacteria. *Nucleic Acids Res.* **1990**, *18* (14), 4296.

(8) Aymanns, S.; Mauerer, S.; van Zandbergen, G.; Wolz, C.; Spellerberg, B. High-Level Fluorescence Labeling of Gram-Positive Pathogens. *PloS One* **2011**, *6* (6), e19822. https://doi.org/10.1371/journal.pone.0019822.

(9) Smith, H. E.; Wisselink, H. J.; Vecht, U.; Gielkens, A. L.; Smits, M. A. High-Efficiency Transformation and Gene Inactivation in Streptococcus Suis Type 2. *Microbiol. Read. Engl.* **1995**, *141 ( Pt 1)*, 181–188. https://doi.org/10.1099/00221287-141-1-181.
